# The healthy workplace initiative

**DOI:** 10.4103/0019-5278.72236

**Published:** 2010-08

**Authors:** S. M. Shanbhag

**Affiliations:** Group Medical Advisor, Reliance Group of Industries, Mumbai, India E-mail: shrinivas.shanbhag@ril.com

In our society, doctors are seen to have reactive (curative) rather than a proactive (preventive) role. All of us believe that a doctor is required only when one is ill. Organizations, which are an integral part of the society, also believed that the medical departments are needed to take care of their injured or ill human resources and hence perform a service function and did not have a meaningful role in the business. We have seen many instances when the medical departments have been axed in austerity drives when the organizations have not been performing well financially.

Historically, organizations have followed a financial model. The healthiness of a business meant sound financial health. Successful organizations were those that maximized monetary profits. This narrow view normally prevents positive health. This thought process was also responsible for creating a divide between the manager and the worker – “the thinker and the doer”. Workers were thought to be machines, which explains the connotation of the term “manpower”. Workers were thought to be dispensable and the issues relating to them were not considered important. It was thought that as they were being compensated for the work performed by them they did not deserve any further attention. Financial returns and rewards were not enough to motivate the workers to sustain high levels of performance. Human beings were different from other resources because they had their own views, thoughts and feelings. Organizations took a long time to realize that beside financial returns, physical well being, intellectual recognition and emotional satisfaction were also important factors in improving performance and productivity.

Organizations started recognizing the above fact in the latter half of the 20^th^ century. Along with various governmental and non-governmental stakeholders, they started initiatives for their employees as well as recognized the need for organizations to interact with the society. These initiatives like corporate governance, corporate social responsibility and learning organizations addressed some issues like good practices and ethics, which had an impact on workers and the society.

The World Health Organization (WHO) defines health as physical, social and mental well being and not merely an absence of disease. The Healthy Workplace initiative, which began in the latter half of the nineties as a WHO initiative, addresses health issues in a holistic manner.

The philosophy of this initiative provides for addressing health issues at the workplace, which include health surveillance, hazard free workplace, health care delivery at the workplaces, support for secondary and tertiary level health care, avenues for satisfying the emotional and spiritual needs of the human being and makes the individual feel that he is a very important part of the organization.

The initiative also addresses the issues of relating to its wider environment by promoting health and wellness in the community and behaving as a responsible corporate citizen of the community’s health, happiness and prosperity.

The implementation of the Healthy Workplace initiative will reduce sickness absenteeism, reduce liability, increase productivity, improve industrial relations and go a long way in image building. The result of all the above is profitability. Hence, making a commitment to this initiative and investing money for it is a recipe for success and value addition to business.

The Healthy Workplace initiative is a partnership between the organization, the unions, the governmental organizations and the NGOs. This initiative has been implemented with a good degree of success in UK, Europe, Canada and Latin America. The global HECONet – Healthy Companies Network – is a non-profit organization working in line with WHO strategies outlined in the Ottawa and Jakarta declarations focused on the occupational setting in the Healthy Work Approach. It acts as a facilitator between the governments and businesses, and the Indian Association of Occupational Health is strategically placed to take an initiative to network with international agencies and bring together other NGOs, governmental agencies and promote this initiative to the managers of progressive industries. Industries implementing this initiative will, without doubt, reap its benefits, as Good Health means Good Business.

